# Wide‐ranging genetic variation in sensitivity to rapamycin in *Drosophila melanogaster*

**DOI:** 10.1111/acel.14292

**Published:** 2024-08-12

**Authors:** Benjamin R. Harrison, Mitchell B. Lee, Shufan Zhang, Bill Young, Kenneth Han, Jiranut Sukomol, Vanessa Paus, Sarina Tran, David Kim, Hannah Fitchett, Yu‐Chen Pan, Philmon Tesfaye, Alia W. Johnson, Xiaqing Zhao, Danijel Djukovic, Daniel Raftery, Daniel E. L. Promislow

**Affiliations:** ^1^ Department of Laboratory Medicine and Pathology University of Washington School of Medicine Seattle Washington USA; ^2^ Ora Biomedical, Inc. Tukwila Washington USA; ^3^ Northwest Metabolomics Research Center, Department of Anesthesiology and Pain Medicine University of Washington School of Medicine Seattle Washington USA; ^4^ Department of Biology University of Washington Seattle Washington USA; ^5^ Jean Mayer USDA Human Nutrition Research Center on Aging Tufts University Boston Massachusetts USA

**Keywords:** *Drosophila*, metabolomics, mTOR, natural variation, rapamycin

## Abstract

The progress made in aging research using laboratory organisms is undeniable. Yet, with few exceptions, these studies are conducted in a limited number of isogenic strains. The path from laboratory discoveries to treatment in human populations is complicated by the reality of genetic variation in nature. To model the effect of genetic variation on the action of the drug rapamycin, here we use the growth of *Drosophila melanogaster* larvae. We screened 140 lines from the *Drosophila* Genetic References Panel for the extent of developmental delay and found wide‐ranging variation in their response, from lines whose development time is nearly doubled by rapamycin, to those that appear to be completely resistant. Sensitivity did not associate with any single genetic marker, nor with any gene. However, variation at the level of genetic pathways was associated with rapamycin sensitivity and might provide insight into sensitivity. In contrast to the genetic analysis, metabolomic analysis showed a strong response of the metabolome to rapamycin, but only among the sensitive larvae. In particular, we found that rapamycin altered levels of amino acids in sensitive larvae, and in a direction strikingly similar to the metabolome response to nutrient deprivation. This work demonstrates the need to evaluate interventions across genetic backgrounds and highlights the potential of omic approaches to reveal biomarkers of drug efficacy and to shed light on mechanisms underlying sensitivity to interventions aimed at increasing lifespan.

## Graphical abstract

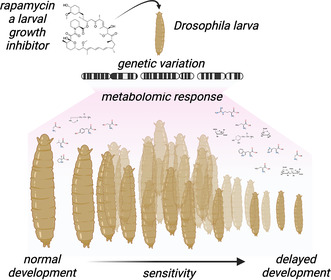

## 1 INTRODUCTION

Rapamycin and related inhibitors of the mechanistic target of rapamycin (mTOR) pathway are of great interest as potential interventions to extend organismal lifespan (Kaeberlein, 2017). Studies of diverse organisms, from yeast to mammals, suggest that decreased mTOR signaling can extend lifespan (Bjedov et al., 2010; Harrison et al., 2009; Kaeberlein et al., 2005). However, laboratory studies of rapamycin have focused almost exclusively on a handful of inbred laboratory strains. As such, little is known regarding variation in the efficacy of rapamycin in natural populations. Studies in yeast (Schleit et al., 2013), flies (Jin et al., 2020), worms (Onken et al., 2022), and mice (Liao et al., 2010) have pointed to a major role for genetic variation in shaping the response to aging interventions. Similarly, two studies have found that rapamycin's effect on *Drosophila* lifespan varies strongly by genotype (Bjedov et al., 2010; Rohde et al., 2021). However, these latter studies were relatively small in scale. Little is known about the full extent of variation in sensitivity to rapamycin, nor the underlying mechanisms for this variation. Given the interest in the potential of rapamycin to increase lifespan, it is critical that we understand the potential causes and consequences of variation in rapamycin sensitivity.

The emphasis on experimental work on a limited number of strains is perhaps not surprising, as lifespan studies are time‐ and labor‐intensive. In the 1990s, researchers found that at least in some species, stress resistance could be used as a proxy for lifespan, saving time and effort (Lithgow & Walker, 2002). In the present work, we were interested in identifying genetic variation for response to rapamycin in the fruit fly, *Drosophila melanogaster*. While our eventual goal is to identify variation in the lifespan response, here we turn our attention to development time, which can be slowed dramatically by rapamycin.

To explore the role of genetic background on response to rapamycin, here we target larval growth. Numerous studies have used the growth of larvae to investigate mTOR function in *Drosophila* (Layalle et al., 2008; Oldham et al., 2000; Scott et al., 2004; Zhang et al., 2000). Larval development is delayed by a variety of manipulations that reduce mTOR activity, including rapamycin and starvation (Oldham et al., 2000; Scott et al., 2004; Zhang et al., 2000). The mTOR pathway regulates larval growth, primarily through its effects on cell growth, but also influences a variety of other organismal processes in *Drosophila* including cell differentiation and development, hematopoiesis, and behavior (Benmimoun et al., 2012; Wen et al., 2017). This diversity of downstream targets provides the opportunity for a sensitive readout of mTOR function in response to rapamycin. Here we used delayed larval development time as a measure of rapamycin sensitivity. To model natural genetic variation, we used the *Drosophila* Genetic Reference Panel (DGRP), a population of wild‐derived inbred lines that captures substantial genetic variation (Huang et al., 2014).

We found remarkable genotypic variation in developmental delay in response to rapamycin across the DGRP, with some genotypes showing rapamycin sensitivity that exceeded common laboratory lines, and others that were completely resistant, showing no effect even at over 500 times the dose that we used for screening. We found weak evidence of heritability that could be attributed to the single nucleotide polymorphism (SNP) genotype data available for the DGRP, with estimates varying substantially in population subsamples. A genome‐wide association study (GWAS) failed to identify statistically significant associations between rapamycin sensitivity and any single genetic variant or gene. However, when we looked for larger sets of variants aggregated by biological pathway, we found associations between rapamycin sensitivity and pathways involved in morphology, development, and cell signaling. To further explore the mechanisms by which genotype affects rapamycin sensitivity, we sampled groups of lines at the extremes of the phenotypic distribution, a design that treated rapamycin sensitivity as a dichotomous trait. Finally, we turned to metabolome profiling as a strategy to bridge the gap between genotype and phenotype for complex, polygenic traits (Harrison et al., 2020), asking whether metabolome profiles were associated with sensitivity using metabolomics.

In the absence of rapamycin, the metabolome of sensitive and resistant lines was indistinguishable. However, within sensitive lines, we observed strong and consistent effects of rapamycin on the metabolome, effects that strikingly recapitulated those of starvation. This work demonstrates both extensive polygenic variation in rapamycin sensitivity, while also showing that sensitive lines appear to converge on a common metabolome response. This work provides a strong rationale for the development of metabolomic biomarkers for rapamycin sensitivity.

## 2 RESULTS

### 2.1 Extensive natural genetic variation in sensitivity to rapamycin

**FIGURE 1:**
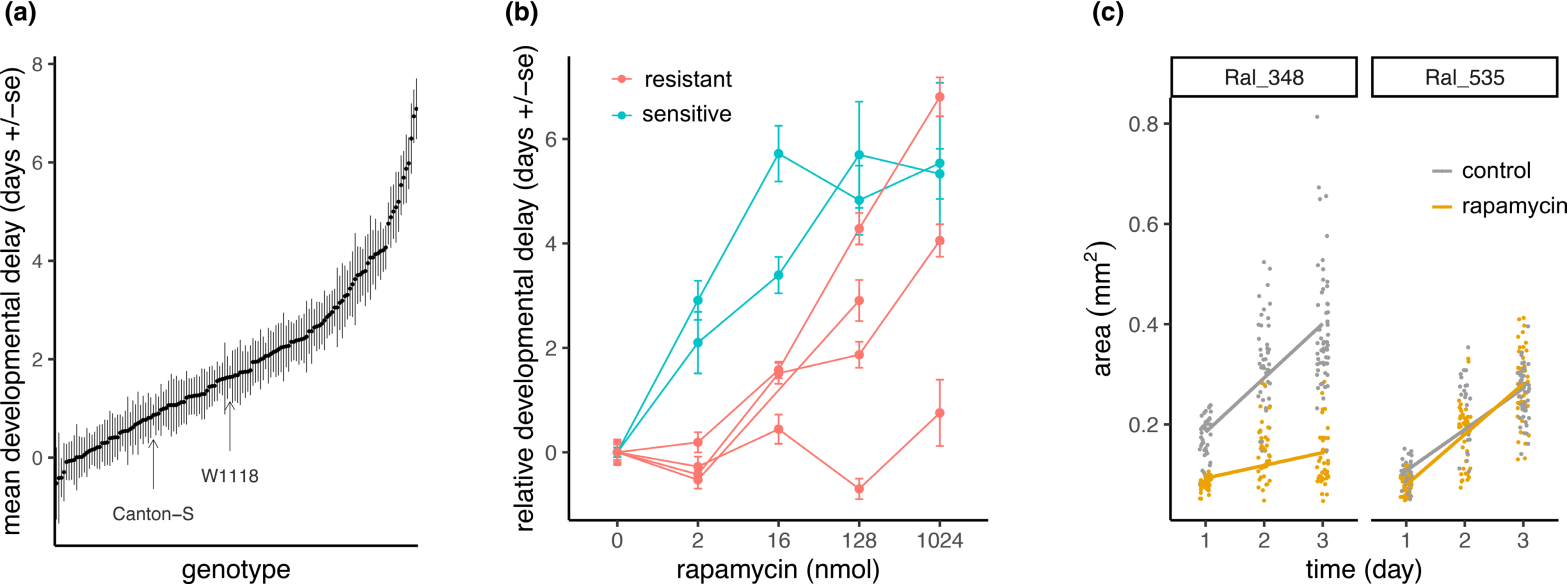
Wide‐ranging genetic variation in rapamycin sensitivity in the *Drosophila* Genetic Reference Panel (DGRP). (a) The mean developmental delay caused by rapamycin (mean pupation time on 2 nmol rapamycin ‐ mean pupation time on control) for each of 140 DGPR lines and two laboratory lines (Canton‐S and w^1118^). Error bars are the pooled standard error of the mean (Section 4). The average number of larvae per condition was 77 (range = 3 to 216). (b) Mean pupation time of four lines that are resistant (red) and of two sensitive lines (blue) at doses of rapamycin ranging from 0 to 1024 nmol. (c) The size of larvae from a representative resistant line (Ral_535, mean delay =0.02 ± 0.40d), and a representative sensitive line (Ral_348, mean delay = 5.5 ± 0.61d) was monitored over the first 3 days of development on either control food (black), or food containing rapamycin (yellow). The size of 17–67 larva were measured each day. Across an additional five lines analyzed, both the size and the rate of size increase were significantly affected by rapamycin in sensitive lines, but not in resistant lines (ANOVA, *p* < 0.05, Figure S2).

Larval growth and the pace of development are mTOR‐dependent and rapamycin‐sensitive, and so we reason that the rate of larval development provides an organismal model to study sensitivity to rapamycin (Oldham et al., 2000; Zhang et al., 2000). To understand how natural genetic variation modifies the response to rapamycin, we measured time to pupation of *Drosophila* embryos that developed on food containing either 2 nmol rapamycin or vehicle control (Section 4). We did so among 140 lines from the DGRP and two standard laboratory strains (W^1118^ and Canton‐S) (Figure 1a, Table S1). Across all lines tested, the average developmental delay, measured as the pupation time difference between rapamycin and control, was 2.0 ± 1.6 days (SD), representing an average delay of 21.3%. We found wide variation in development delay across the DGRP with some lines showing insignificant delay, and others where mean development was delayed up to 7.1 days (69.1%) (Figure 1a). While pupation time on control food varied across the DGRP, it was not correlated with developmental delay on rapamycin (Spearman's *ρ* = −0.133, *p* = 0.115, Figure S1).

To explore the degree to which rapamycin sensitivity is influenced by dose, we sampled lines from the extremes of the phenotypic distribution to obtain four relatively resistant lines (mean delay of −0.058 ± 0.30 days (SD)), and two sensitive lines (mean delay of 6.4 ± 0.75 days (SD)). We measured their development time on rapamycin doses ranging from 2 to 1024 nmol. Both sensitive lines showed increasing delay beyond 2 nmol. However, the four resistant lines showed various responses to higher doses of rapamycin, with three of the four resistant lines delayed above 2 nmol, and one showing no evidence of delay, even when treated at 1024 nmol (Wilcoxon rank sum test, *p* < 0.05, Figure 1b).

To test whether delayed development was associated with effects on larval size, we sampled four sensitive lines with a mean developmental delay of 6.21 ± 0.63 days (SD), and two resistant lines with a mean developmental delay of −0.022 ± 0.05 days (SD). Measuring the size of larvae of these lines over the course of 3 days revealed a significant effect of rapamycin on larval size (ANOVA, size × treatment × phenotype, *F*_(1,1391.44)_ = 135.3, *p* = 6.6 × 10^−30^), and on growth rates among the sensitive, but not the resistant lines (size × day × treatment × phenotype *F*_(2,1391.44)_ = 25.0, *p* = 2.1 × 10^−11^, Figure S2, Figure 1c, Section 4). Moreover, there was no effect of rapamycin on larval size at any day among the two resistant lines (*F*_(1,511.08)_ = 0.97, *p* = 0.33, Figure S2).

### 2.2 Genetic variation for rapamycin sensitivity maps to biological pathways

**FIGURE 2:**
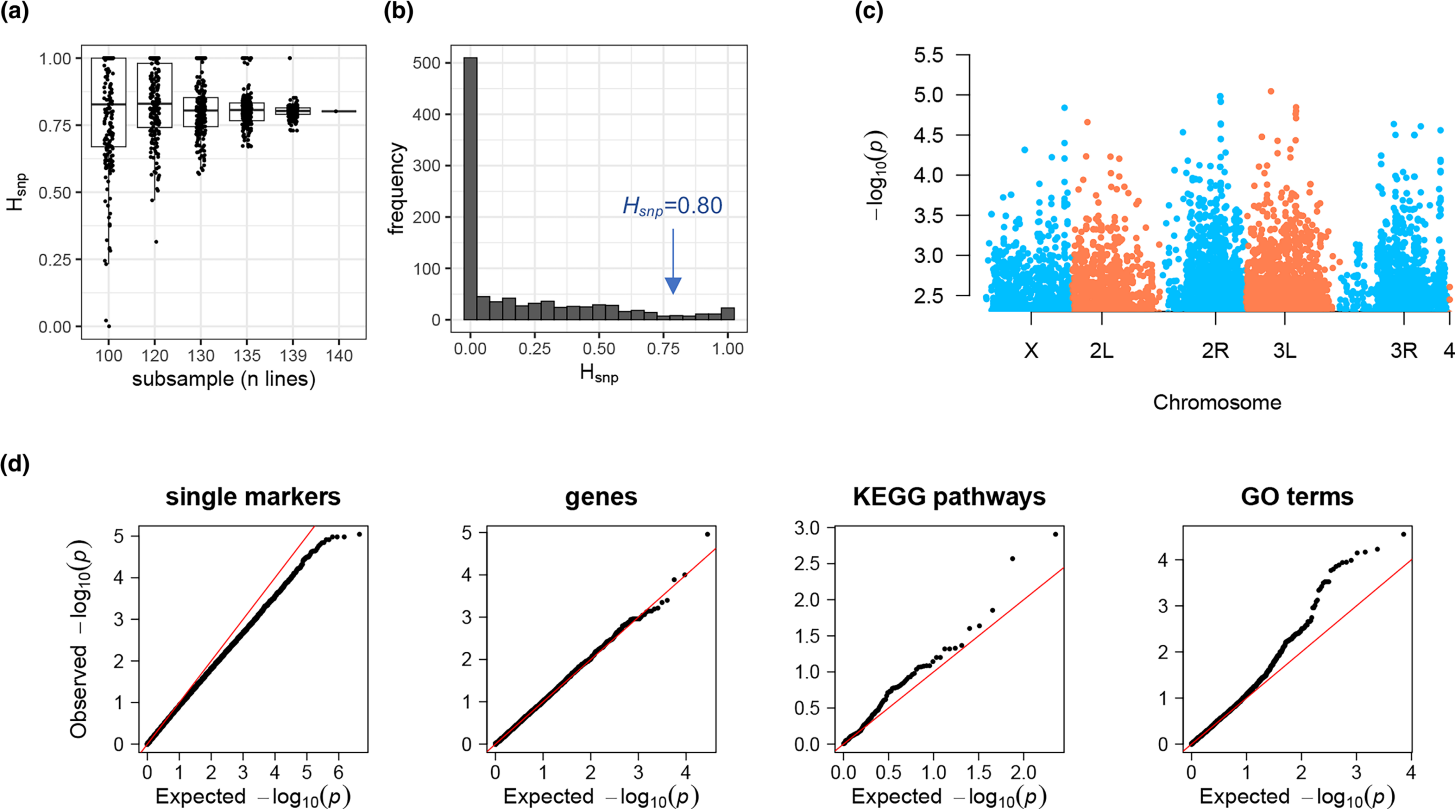
Genetic variation in rapamycin sensitivity maps to biological pathways. (a) SNP heritability (*H* ^2^_SNP_) estimated by gBLUP among the 140 DGRP lines was 0.802 ± 0.35(SE). *H* ^2^_SNP_ estimates in each of 200 bootstrap subsamples of n lines. (b) *H* ^2^_SNP_ estimated in each of 1000 permutations of the phenotype are highly zero‐inflated and give an empirical *p* = 0.041 for the real estimate (arrow). (c) There was no single marker associated with rapamycin sensitivity at the genome‐wide threshold (CVAT, FDR >0.99 for all markers). Manhattan plot showing the –log_10_ *p* value of 10,854 genetic markers (*p* ≤ 0.01), across each chromosome arm. The remaining 1.8 × 10^6^ markers (*p* > 0.01) are not plotted. (d) Genetic covariance can be partly attributed to genetic markers aggregated by Kyoto Encyclopedia of Genes and Genomes (KEGG) pathways or gene ontology (GO) terms. Q‐Q plots for the –log_10_ *p* values for individual markers, and the empirical *p‐*values among the CVAT analyses of genome features representing genes, KEGG pathways and GO terms.

To explore the potential association between genetic variation in the DGRP and the substantial variation in rapamycin sensitivity, we measured SNP heritability (*H* ^2^_SNP_), the proportion of variance explained by the genetic relatedness of the DGRP lines as estimated by the genotype data, using the gBLUP method (Rohde et al., 2020; Section 4). In the full dataset of 140 lines, *H* ^2^_SNP_ = 0.80 (±0.35 SE), with large error in the estimate likely reflecting the modest power of 140 lines (Figure 2a). Bootstrap sampling of smaller numbers of lines indicated that approximately 130 lines was a sufficient number to estimate the *H* ^2^_SNP_ observed in the complete data set, and subsamples below 130 lines showed wide variation in *H* ^2^_SNP_ (Figure 2a). Given the relatively large variation in *H* ^2^_SNP_, we further tested the covariation between rapamycin sensitivity and the genetic relatedness of the DGRP by permutation. Permuted heritability estimates were typically near zero, and *H* ^2^_SNP_ exceeded the estimate from real data in only 41 of 1000 permutations (*p* = 0.041, Figure 2b).

To look for genetic markers that might explain variation in rapamycin resistance we estimated effects of 1.09 × 10^6^ markers using a linear mixed model (Section 4). We failed to identify single markers that reached genome‐wide significance (*p* > 9 × 10^−6^, FDR >0.999, Figure 2c, Table S2). We also asked if rapamycin sensitivity is associated with the mitochondrial polymorphisms in the DGRP, including single marker analysis or associations with the three main mitochondrial genotypes among these 140 lines. However, no mtDNA markers associated with sensitivity to rapamycin (Supplementary Materials).

The failure of individual markers to explain much of the phenotypic variation indicated that sensitivity may be highly polygenic, involving many variants, each of weak effect. While a small effect of an individual variant may not lead to differences among lines, perhaps several such small effects could be shared among a gene, or among the genes in a pathway. If so, the collective variation in a pathway then could associate with phenotypic variation among lines (Edwards et al., 2016). This idea prompted us to use the covariance association test (CVAT) (Rohde et al., 2016) to ask whether markers that were aggregated at the gene‐level associated with rapamycin sensitivity. The CVAT approach estimates the covariance between genome‐wide genetic effects and genetic effects of a subset of markers, and CVAT can be used to test whether markers associated with a gene, or other genome feature, are effectively enriched for associated variants. When applied to the 1–4471 (mean = 596) markers within each of 14,125 genes, we found the set of markers within the gene *DptA* was weakly associated with rapamycin sensitivity at an FDR of <16% (*p* = 1 × 10^−5^, Table S3). We then extended this analysis to include markers within ±1 kb of the primary transcript of each gene, which incidentally included 830 additional genes that then had enough markers for this analysis. Variation at *DptA* ± 1 kb was less associated with sensitivity (*p* = 4 × 10^−5^, FDR <0.64), and this analysis did not reveal any additional genes (Table S3).

We then applied CVAT at the level of biological pathways, testing variation in the genes in each of 127 Kyoto Encyclopedia of Genes and Genomes (KEGG) pathways, and 4346 gene ontology (GO) terms. While no terms were significant at FDR = 5%, we found two KEGG pathways and 23 GO terms significant at FDR <0.20 (Table S3). Associated pathways include TGFβ and MAPK signaling, hemocyte development, nurse cell apoptosis, and regulation of tracheal diameter. We note that KEGG pathways and GO terms are not mutually exclusive. In fact, the TGFβ signaling pathway as represented by both KEGG (dme04350) and GO (GO:0060391) were associated with rapamycin sensitivity (Table S3). While the mTOR signaling pathway was not itself significant (*p* = 0.03, FDR = 0.528), the MAPK pathway is partially nested within the mTOR pathway. The null distribution of *p‐*values is expected to be uniform. Using Q‐Q analysis against the null distribution of *P*, both KEGG pathways and GO terms seem to associate more strongly with rapamycin sensitivity when compared to associations of single genetic markers, or variation in individual genes (Figure 2d).

### 2.3 Rapamycin reshapes the metabolome of sensitive larvae

**FIGURE 3:**
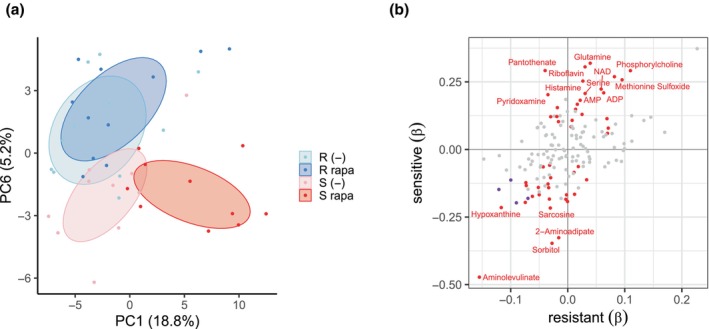
Rapamycin primarily affects the metabolome of sensitive lines. (a) Plot of PC_6_ over PC_1_ of 154 metabolites in larvae of six resistant (*R*) and seven sensitive (S) lines on food treated with either rapamycin (rapa) or control (−), depicts the distinction between *R* and S along PC_6_, and the effect of rapamycin particularly on the metabolome of the S lines on PC_1_. Ellipses are 60% confidence intervals. (b) The effect of rapamycin (*β*) on metabolites in resistant and sensitive lines. Metabolites with effects at FDR ≤5% in sensitive lines (red) or both sensitive and resistant lines (purple). No metabolites were affected in resistant lines that were not also affected in sensitive lines. Metabolites not affected are in grey. The correlation between *β* in resistant and sensitive lines is significant (Spearman's *ρ* = 0.48, *p* = 4.0 × 10^−10^).

To gain further insight into the effect of rapamycin, we analyzed a panel of 154 aqueous metabolites in larvae of six resistant lines, with mean developmental delays ranging from −0.41 to 0.02 days, and seven sensitive lines, with mean delays ranging from 4.76 to 7.09 days, each of which was treated with either rapamycin or control food for 2 days after egg laying. Principal components analysis (PCA) detected an effect of rapamycin on the metabolome among the sensitive genotypes along PC_1_, as well as a distinction between resistant and sensitive lines along PC_6_ (Figure 3a). We failed to identify any individual metabolites associated with the resistance phenotype alone (ANOVA, FDR >5%). However, given the response of sensitive lines to rapamycin (Figure 3a), we looked specifically for metabolites that have treatment effects within resistant or sensitive lines. We fit a mixed model with a fixed treatment effect to the metabolome data from resistant or sensitive larvae. The sign of treatment effects among resistant and sensitive lines was highly correlated (*ρ* = 0.48, *p* = 4.0 × 10^−10^, Figure 3b). However, the magnitude and significance of these effects differed markedly between resistant and sensitive lines. Of the 154 metabolites, four were significantly reduced in the rapamycin condition in both resistant and sensitive lines. However, in sensitive lines, an additional 24 were less abundant and 24 more abundant (FDR <5%, Figure 3b). These results indicate that rapamycin affects metabolome profiles, primarily within sensitive lines, where perhaps the same response is attenuated among resistant lines.

We then sought to identify biochemical pathways whose activity could explain the effects of rapamycin treatment among the resistant or sensitive lines. With 154 metabolites, we were underpowered to perform standard enrichment analysis on the majority of biological pathways, and so we made use of a network enrichment analysis that analyzes nodes in biological networks, looking for those that connect more closely to a set of metabolites than expected by chance (Picart‐Armada et al., 2018). The metabolites affected by rapamycin in both resistant and sensitive larvae—cytosine, guanosine, inosine, and histidine—enrich nucleotide metabolism (dme01232, FDR = 0.002), protein export (dme03060, FDR = 0.017), and purine metabolism (dme00230, FDR = 0.026). When we analyzed the pathways enriched by the 52 metabolites that were affected by rapamycin in sensitive lines, we identified dme00970—aminoacyl‐tRNA biosynthesis (FDR = 0.003).

### 2.4 Rapamycin causes a starvation‐like metabolome in sensitive larvae

**FIGURE 4:**
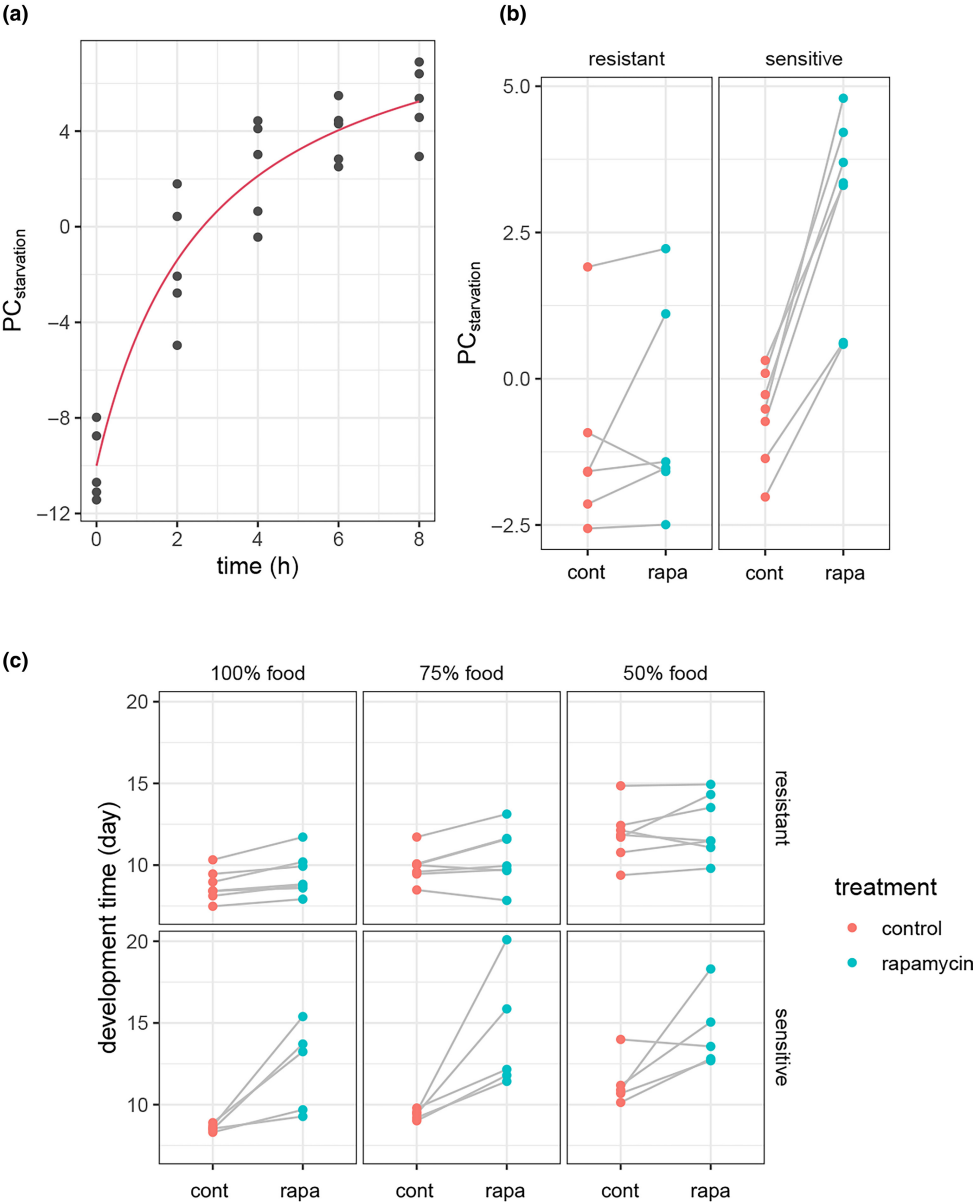
Rapamycin induces a starvation‐like metabolome in sensitive larvae, and sensitivity is unaffected by nutrient deprivation. (a) The first principal component (PC_starvation_) of 84 metabolites in w^1118^ larvae exposed to PBS‐soaked paper for 0–8 h (starvation time, Jouandin et al., 2022). The red line is the fit of an exponential model (*r* ^*2*^ = 0.91, *p* = 9.68 × 10^−6^, Section 4). (b) The metabolome data from the same 84 metabolites in larva of sensitive or resistant lines on control (cont) or rapamycin (rapa) food was projected onto PC_starvation_. Rapamycin had a significant effect on PC_starvation_ that was specific to the sensitive larva (*p* = 8.0 × 10^−5^). (c) The mean pupation times of five sensitive lines and seven resistant lines on control food (100%) or food with the yeast and sugar components diluted to 75% or 50%, were measured under rapamycin and control treatments. Food dilution slowed development (*β*_75_ = 1.09, *p* = 2.2 × 10^−15^; *β*_50_ = 3.02, *p* < 2 × 10^−16^), as did rapamycin treatment among the sensitive lines (*p* < 2 × 10^−16^). However, food dilution did not affect rapamycin sensitivity (treatment × food, and treatment × food × phenotype, *p* < 0.13).

Two related mechanisms that influence larval sensitivity to rapamycin are autophagy and starvation (Jouandin et al., 2022; Scott et al., 2004). In sensitive larvae on rapamycin, we saw increased abundance of metabolites enriching aminoacyl‐tRNA synthesis, and aminoacyl‐tRNA synthases regulate autophagy in larvae (Arsham & Neufeld, 2009). Starvation occurs in low‐nutrient conditions, where mTOR stimulates autophagy to recycle macromolecules back onto the nutrient pool (Scott et al., 2004). We therefore asked if rapamycin treatment has a similar effect to that of food deprivation on the larval metabolome, and if the response in rapamycin‐sensitive larvae is like starvation. Jouandin et al. (2022) measured the aqueous metabolome of larvae of the *Drosophila* line W^1118^ that were deprived of food for up to 8 h (Section 4). Their data included measurement of 84 of the 154 metabolites that we measured here. The first principal component of these 84 metabolites in their data was highly associated with food deprivation time, such that longer starvation led to larger values of PC_1_, which for simplicity, we refer to as PC_starvation_ (Figure 4a). When we used the loadings of the 84 metabolites in PC_starvation_ to project the metabolome data from our study, we find not only that rapamycin treatment led to larger PC_starvation_ values (ANOVA, treatment *F*_(1,27)_ = 40.44, *p* = 1.4 × 10^−6^), but also that there was a highly significant and consistent displacement of the metabolome along the PC_starvation_ axis in rapamycin‐sensitive lines (Figure 4b, ANOVA, treatment × phenotype *F*_(1,27)_ = 21.06, *p* = 9.18 × 10^−5^). Thus, the metabolome of rapamycin‐sensitive larvae, when treated with rapamycin, resembles that of starved larva.

mTOR signals growth and development in response to nutrient status. Thus, we asked if nutrient deprivation would modify sensitivity to rapamycin. We measured the effect of rapamycin on development of seven resistant lines and six sensitive lines, on food containing 75% or 50% of the yeast and sugar of the standard (100%) food. While food dilution delayed development by ~1 day at 75%, and ~2d at 50% (*p* < 2 × 10^−16^), and rapamycin significantly delayed the development of sensitive lines at all tested food levels (*p* = 3.2 × 10^−12^), there was no indication that food dilution modified the sensitivity to rapamycin of either the sensitive of resistant genotypes (food condition × rapamycin treatment; *p* > 0.27; Figure 4c). To test the prediction that development in rapamycin‐sensitive genotypes is inherently more sensitive to nutrient deprivation, we compared the developmental delay elicited by food dilution in sensitive and resistant genotypes in the absence of rapamycin and found no difference in their response (*p* > 0.46; Figure 4c).

## 3 DISCUSSION

Extensive laboratory studies have identified mTOR as a driver or modulator of pathways that are intimately connected to life history and longevity. However, efforts to translate discoveries from laboratory models into medical interventions will inevitably need to address the possibility that the efficacy of these treatments might depend on genetic background. Here we exploit the pace of early *Drosophila* development to examine the influence of genetic variation on the effect of rapamycin. While not directly assessing longevity, focusing on larval development as an indicator of rapamycin activity provided a means to rapidly screen many conditions (Lithgow & Walker, 2002; Scott et al., 2004).

Until now, much of the published work dissecting rapamycin action on lifespan in adult flies has involved the outbred strain *Dahomey* (Bjedov et al., 2010; Schinaman et al., 2019). Using six lines from the DGRP, Rohde et al. (2021) demonstrated genetic variation for the effect of rapamycin on adult longevity, suggesting that genetic variation in part determines sensitivity to this longevity intervention. We show that, at a dose sufficient to delay the development of most *Drosophila* lines, there is a continuum of responses manifesting as developmental delays of −4.5%–79% of the control developmental period. We also show that this range of delay over the same dose is only one axis of variation. Among lines resistant to the screening dose, there is yet more variation in the dose–response, including one of four lines that showed no delay on media containing up to 512× the screening dose, and various degrees of dose–response in the other lines. The developmental delay that we observe was accompanied by slower growth in larval size, consistent with studies that use either pharmacological or genetic manipulation of mTOR activity (Layalle et al., 2008; Oldham et al., 2000; Scott et al., 2004; Zhang et al., 2000). Together our results indicate that *Drosophila* populations maintain substantial natural genetic variation for sensitivity to rapamycin.

### 3.1 How are resistant and sensitive larvae different?

We investigated the variation in rapamycin sensitivity in two ways; first, by looking for genetic variation associated with resistance measured as a continuous trait among 140 DGRP lines, and second, by comparing the metabolome of groups of lines at the extremes of the distribution of resistance and sensitivity. This latter approach has been used successfully to dissect the response of adult flies to hydrogen peroxide, where it revealed remarkable convergence on glycogen metabolism in sensitive genotypes, in contrast to the relative insensitivity of resistant genotypes (Harrison et al., 2020). We find a similar pattern in the response to rapamycin as well. In contrast to the effect of rapamycin on the metabolome of resistant larva, the metabolome of sensitive larvae shows a dramatic response with respect to many metabolites. This analysis considered resistant and sensitive lines as binary classes and so was underpowered to detect variation within the two classes of resistant and sensitive lines.

In sensitive larvae under rapamycin, we see effects on amino acids, including glutamine, serine, histidine, and glutamic acid, and the fatty acid‐related metabolites phosphorylcholine, acetylcarnitine, and carnitine. These metabolites have peripheral roles in the activity of the TCA cycle, which harvests energy from fatty acids in response to mTOR inhibition (Jouandin et al., 2022). We also show that rapamycin treatment induces a starvation‐like state in the larval metabolome of sensitive larvae (Figure 4), a response that is consistent with transcriptome analysis of mammalian cells treated with rapamycin (Peng et al., 2002). The metabolites affected by rapamycin in sensitive larvae are enriched for the tRNA biosynthesis (tRNA charging) pathway. tRNA charging is the attachment of amino acids to their cognate tRNAs. There is compelling evidence for the involvement of tRNAs in the regulation of larval growth by mTOR (Rideout et al., 2012; Rojas‐Benitez et al., 2015), and so this mechanism may explain the sensitivity of lines in this study. Alternatively, the enrichment of tRNA charging may simply reflect the numerous amino acids whose abundances are affected by rapamycin. This result is consistent with a shift from protein synthesis and toward autophagy under mTOR inhibition (Scott et al., 2004).

The metabolomic response of sensitive larvae to rapamycin indicates that they either perceive nutrient limitation via reduced mTOR signaling or are under actual nutrient limitation by some undetermined mechanism. Nutrient supply and the perception of its status is critical for growth and the developmental transitions that determine the time it takes to progress from egg to pupa (Texada et al., 2020). We hypothesized that, by experimentally limiting nutrients, larvae that were otherwise insensitive would become sensitive due to reduced growth signaling. Instead, we found that limiting nutrients to levels sufficient to delay development was not sufficient to alter the sensitivity of larvae to rapamycin, neither making resistant larvae sensitive, nor enhancing the sensitivity of sensitive larvae (Figure 4c). The independence of the developmental delay induced by rapamycin and that from nutrient limitation suggests that either the starvation‐like effect of rapamycin on the metabolome is independent or downstream of the developmental delay or that food dilution acts independently on development.

The ultimate cause of variation in rapamycin sensitivity that we describe is genetic. Relatedness among the DGPR, as reflected in their similarity over ~10^4^ genetic markers, accounts for approximately 80% of the variance in the developmental delay among the lines (Figure 2). Such a high *H* ^2^_SNP_ suggests rapamycin sensitivity is highly predicted by the genome‐wide similarity among lines. While this may be the case, we also show that this estimate is sensitive to the number of lines analyzed and, while significantly non‐zero, is only weakly significant based on permutation tests (*p* = 0.041, Figure 2). While we were not able to find individual genetic markers associated with rapamycin sensitivity, markers grouped at the gene and pathway levels identified several putative modifiers of rapamycin sensitivity. Our observation of stronger associations when genetic variation was aggregated to the gene or pathway levels (Figure 2d) is similar to results from previous studies (Edwards et al., 2016). At a marginal FDR threshold of <16%, variation in *DptA*, which encodes an antimicrobial peptide (AMP), associates with rapamycin sensitivity. In this study, larval growth was measured on media containing the fungicide tegosept and the antibiotics kanamycin and tetracycline (Section 4), conditions that substantially reduce microbial load, so the association between rapamycin sensitivity and *DptA* is not likely due to infection. Independent of infection, *DptA*, along with other AMPs, is induced by FOXO in response to nutrient stress, or in response to mTOR inhibition by rapamycin, and their induction is required for timely larval development (Kamareddine et al., 2018; Varma et al., 2014). Therefore, the genetic association we find provides suggestive evidence for a causal role for *DptA* in the growth effect of rapamycin.

At the pathway level, genetic variation in rapamycin sensitivity associates with developmental processes, including embryonic hindgut morphogenesis, foregut morphogenesis, and TGFβ signaling (Table S3). In adult flies, rapamycin affects the activity of intestinal stem cells, and the intestine is a target for lifespan extending effects of rapamycin (Schinaman et al., 2019). Thus, genetic variation in gut development or function may explain some of the variation in sensitivity to rapamycin that we report. In larvae, *Dpp*, a fly homolog of the growth‐regulatory ligand TGFβ, modulates mTOR activity (Denton et al., 2019). While not significant genome‐wide (*p* = 10^−4^, FDR = 61%), *Dpp* is the second‐most associated gene in our analysis. The association between rapamycin sensitivity and TGFβ signaling lends support to a non‐canonical model of rapamycin action that has emerged from other studies. In the canonical mammalian models, rapamycin binds to the FK506‐binding protein FKBP12, and this complex binds mTOR, preventing its function (Liu & Sabatini, 2020). The *Drosophila* homolog of FKBP12 is part of an eight gene family, *Fkbp12* being the closest homolog to human FKBP12 (Ghartey‐Kwansah et al., 2018). While the interaction with rapamycin, FKBP12, and mTOR has not been demonstrated in flies, there is evidence that rapamycin can interfere with a direct interaction between FKBP12 and the Dpp receptor (Chen et al., 1997; Wang et al., 1996). Together with the genetic association we find, these results suggest that rapamycin may have direct cellular targets other than FKBP12‐mTOR that are relevant for growth in *Drosophila* (Chen et al., 1997; Miyakawa et al., 2018). We did not find any association with variants in the Tor gene, and only weak association with genetic variation in the mTOR pathway (Table S3). However, the association with MAPK signaling indicates an axis of variation that intersects with the mTOR pathway.

Given that rapamycin has well‐defined targets, it is somewhat surprising that the heritable genetic signal appears to be diffused over many loci, failing to associate strongly with any of the many thousands of markers in the DGRP (Huang et al., 2014). This could be due either to associations with alleles that did not meet our minor allele frequency threshold, to a lack of segregating variants in mTOR pathway‐associated genes, or to latent genetic variation that at the time of the last public data release (Freeze 2.0) was either undetermined or remained heterozygous (Huang et al., 2014). The mapping we describe only incorporates markers that were called homozygous in Freeze 2.0, and so would not consider loci that have since fixed and that may associate with sensitivity.

Beyond the implications of demonstrating a high degree of genetic variation for the response to a longevity intervention, this work points toward a strategy to detect, predict, and explain variation in the response to rapamycin in *Drosophila* and other species. While the genetic signal captured here is either highly polygenic, or unresolved, we show here that the metabolomic signal is strong and consistent. Future efforts can and should investigate the utility of the metabolome as a biomarker of the response to rapamycin or other pharmacological healthspan interventions.

### 3.2 Limitations of the study

The current study measures developmental delay at a dose of rapamycin that reveals substantial variation across genotypes. We also show a dramatic dose–response among lines, so perhaps phenotypic variation in the response to an alternative dose might associate more strongly with underlying genetic variation. We also focus entirely on larvae and so how the variation in the larval response relates to the variation described in the effect of rapamycin on adult fly lifespan is an open question (Bjedov et al., 2010; Rohde et al., 2021). The effect of rapamycin on adult *Drosophila* lifespan is also sex‐dependent (Bjedov et al., 2010). Because determining the sex of larvae is laborious, rapamycin sensitivity was measured in mixed sex populations and therefore some portion of the phenotype may be due to sex bias among the larvae surveyed per line per condition.

## 4 METHODS

### 4.1 Flies

The Canton‐S stock and lines from the DGRP were purchased from the Bloomington *Drosophila* Stock Center (Indiana University, Bloomington, IN). W^1118^ was kindly gifted by Leo Pallanck, University of Washington. For routine propagation, flies were cultured on media containing 5.5% dextrose, 3% sucrose, 6% corn meal, 2.5% yeast, 0.9% agar, 0.9% EtOH, 0.3% tegosept, and 0.3% propionic acid. *Drosophila* populations were maintained at ~200 eggs per bottles, with ~50 mL media, and cultured under a 12:12 h light: dark cycle at 25°C with 40%–60% humidity.

### 4.2 Media preparation

To increase batch‐to‐batch consistency for the developmental timing screen and minimize bacterial growth during the assay, we used a modified media recipe. Experimental media consisted of 6% dextrose, 3% sucrose, 6% corn meal, 2.5% yeast, 0.9% agar, 1.2% EtOH, 0.3% tegosept, 0.3% propionic acid, 50 μg/mL kanamycin, and 20 μg/mL tetracycline. To make experimental media, 120 g corn meal, 50 g yeast, and 18 g agar were gently boiled in 2 L diH_2_O on a heated stirrer, and then autoclaved at 121°C for 45 min. Media were returned to stirrer, allowed to cool to 60°C, and then 220 mL of 50% dextrose, 120 mL 50% sucrose, 6 mL of propionic acid, and 6 g tegosept dissolved in 24 mL ethanol, were added. Finally, 50 μg/mL kanamycin and 20 μg/mL tetracycline were added. A peristaltic pump was used to dispense 10 mL of media into vials which were then stored at 4°C and used within 2 weeks of preparation. For diluted yeast and sugar food, the above protocol was followed, but the amount of yeast, glucose, and sucrose was reduced to 75% or 50% of the original amounts while other ingredients remained the same.

Rapamycin treatment was administered as an overlay to food in vials. To prepare rapamycin and control media, either a 1 mM rapamycin stock was made by dissolving rapamycin in EtOH at room temperature and then kept at −20°C; or, for the dose–response experiments, 1 mL of 20.5 mM rapamycin stock was made by dissolving 18.7 mg to 1.0 mL in EtOH by vortex mixing in a 1.5 mL tube. Rapamycin stocks were diluted to the appropriate concentration with EtOH, and control solution was just EtOH. For all treatment and control vials, 50 μL of solution was overlaid onto vials, and vials were covered and left overnight on the benchtop to allow food surface to dry.

### 4.3 Development time assay

To collect embryos for development time analysis, 200–300 flies from each line were allowed to lay eggs on grape juice agar plates in egg chambers, with a small quantity of yeast paste and diluted apple cider vinegar on each plate. Fly populations were maintained in egg chambers for 2 to 3d and plates were replaced each day. On the day before embryo collection, plates were replaced in the evening and embryos were collected the next morning to randomly numbered experimental vials (40 embryos per vial) using platinum wire picks that were flame‐sterilized between vials, with three to four vials per treatment condition. Vials were kept in trays at 25°C, 12/12 h light cycle and 50%–60%RH. The DGRP lines were screened in batches of ~10–30 lines each. To estimate batch‐to‐batch variation, two DGRP lines (Ral45 and Ral321) and two laboratory‐adapted lines (W^1118^ and Canton‐s) were tested in each batch. To verify the genotype of experimental flies, adults from egg chambers were stored at −20°C until PCR genotyping. Development times were recorded when new pupae were counted and removed from each vial, once or twice per day, until all vials ceased to produce pupae for two consecutive days.

Mean pupation time for each line treated with rapamycin or vehicle was estimated by pooling all pupae across replicates (mean = 77, range = 3–216 per genotype and condition). To quantify the developmental delay due to rapamycin, the average pupation time for the control condition was subtracted from the average pupation time on rapamycin. To measure the error in the estimated developmental delay for each line, we calculated the pooled standard deviation (*s*) to then get the pooled standard error (SE).

$$\text{pooled}\;s=\sqrt{\;\frac{\left(n_{r}-1\right)s_{r}^{2}+\left(n_{c}-1\right)s_{c}^{2}}{n_{r}-n_{c}-2}}$$

Where *n*_*r*_ and *n*_*c*_ are the numbers of pupae in rapamycin and control vials respectively and *s*_*r*_ and *s*_*c*_ are the respective standard deviations of development times in each condition. Pooled standard error is then:

$$\;\text{pooled}\;\mathrm{SE}=\sqrt{\text{pooled}\;s\times \sqrt{\frac{1}{n_{r}}+\frac{1}{n_{c}}\;}}$$

### 4.4 Larval size

To measure effects of rapamycin on larval size, we raised larvae from embryos on food with and without rapamycin and collected 17–67 (mean = 39) larvae from each genotype and condition on each of Days 1, 2, and 3. Larvae were collected into phosphate‐buffered saline (PBS) on microscope slides, heat‐killed on a hot block at 70°C for ~1 min and imaged under a microscope. The size (area, mm^2^) of each larva was measured by a single blinded researcher who traced the outline of larvae, calculated the area in pixels using ImageJ, and converted to mm^2^ (Schneider et al., 2012). To test the hypothesis that sensitivity phenotype influences the effect of rapamycin on size and/or growth rate, we used a linear mixed model that included a random effect of line (1|line), and two‐way and three‐way interaction terms (e.g., *β*_day×T×P_) between effects of rapamycin treatment (*β*_T_ = rapamycin vs. control), larval area and growth rate (area by *β*_day_), and sensitivity phenotype of the sampled genotypes (*β*_P_ = resistant vs. sensitive).

$$\text{area}\sim \beta_{\mathrm{day}}+\beta_{T}+\beta_{P}+\beta_{\mathrm{day}\times T}+\beta_{T\times P}+\beta_{\mathrm{day}\times P}+\beta_{\mathrm{day}\times T\times P}+1|\text{line}+\varepsilon$$

### 4.5 Genetic association

The developmental delay phenotype for each line was prepared for association testing by normalizing and correcting for batch effects across the DGRP screen. This was done by first adding one (to remove negative values), and then applying the Box‐Cox transformation. Transformed data were then centered within each experimental batch by subtracting the mean values of the four lines included in every batch (Ral45, Ral321, W^1118^, and Canton‐S). The centered data were then scaled within each batch by dividing by the batchwise standard deviation.

SNP heritability (*H* ^2^_SNP_) was estimated using the proportion of variance in rapamycin sensitivity (*y*) that was explained by the joint effects of all markers, the genomic best linear unbiased predictions (gBLUPs). The gBLUPs were estimated as a random effects (*v*) of the genomic relationship matrix (GRM, Z), in a mixed model that included fixed effects (*β*) of Wolbachia status and the genotype of inversions In.2 L.t, In.2R.NS, In.3R.P, In.3R.K, and In.3R.Mo in a design matrix (*X*), and Gaussian error (*ε*) using the qgg package (Rohde et al., 2020).

y=Xβ+Zυ+ε

The GRM was built using the grm function in the qgg package, on mean‐centered and scaled genotypes of 6.5 × 10^4^ variants among the 140 lines in the study (MAF ≥5%, genotyping rate ≥95%, and LD‐pruned to *r* ^2^ ≤ 0.8 in 200 kb windows, sliding 5 kb, with PLINK 1.9 (Chang et al., 2015)).

Genetic associations with rapamycin sensitivity were conducted in two ways, by single marker analysis, and by a set‐based approach using the covariance association test (CVAT) (Rohde et al., 2016) to look for gene‐ and pathway‐level associations. To get single marker effects, the covariance between each of 1.09 × 10^6^ genetic markers (MAF >5%, genotyping rate >80%) and the gBLUPs was tested using the lma function in the qgg package. Genetic markers included 1.01 × 10^6^ snps, 4.3 × 10^4^ deletions, 3.4 × 10^4^ indels, and 937 multiple nucleotide polymorphisms. The resulting *p‐*values were adjusted for multiple testing using the FDR approach (Storey & Tibshirani, 2003). To test for gene‐ and pathway‐level associations we used the CVAT method. At the gene level, each genome feature that we tested included either all markers located within the primary transcript of a gene (based on flybase 5.57 annotations, flybase.org), or, in a separate analysis, the features also included markers ±1 kb from the primary transcript. At the pathway level, gene‐level features were further combined into GO terms and KEGG pathways. We removed pathways with only a single gene or with fewer than 200 total markers, and then one pathway at a time, all markers in all genes were fit simultaneously (Rohde et al., 2016). The test statistic in CVAT has an undefined probability distribution and therefore its significance was evaluated by 1 × 10^6^ permutations within the gsea function, to calculate an empirical *p‐*value. Empirical *p‐*values were then adjusted by FDR.

### 4.6 Metabolomics sampling

For larval metabolomics, embryos from six resistant and seven sensitive lines were harvested from egg chambers and added to replicate rapamycin or control vials as in the developmental screen. For larvae sampling, the egg laying window for flies to deposit embryos on egg chamber plates was reduced to 4–6 h and each line and treatment combination was sampled in three replicate vials. After 2 days in treated vials, 2–3 mL of 1X PBS was added to vials. After 2–4 min, larvae suspended in PBS were pooled between replicates into a petri plate. For each condition, up to 50 larvae were then transferred to 1.5 mL microfuge tubes. Residual PBS was aspirated, and larvae were flash frozen in liquid nitrogen and stored at −80°C until processed for metabolomics. All steps of this experiment were repeated 1 day later, and each replicated experiment is referred to as a batch.

Aqueous metabolites for targeted LC–MS profiling of 54 fly larvae samples were extracted using a protein precipitation method similar to the one described elsewhere (Meador et al., 2020). Samples were first homogenized in 200 μL purified deionized water at 4°C, and then 800 μL of cold methanol containing 124 μM [6‐^13^C] glucose and 25.9 μM [2‐^13^C] glutamate was added (^13^C labeled internal standards were added to the samples in order to monitor sample prep). Afterwards, samples were vortexed, stored for 30 min at −20°C, sonicated in an ice bath for 10 min, centrifuged for 15 min at 18,000×*g* and 4°C, and then 600 μL of supernatant was collected from each sample. Lastly, recovered supernatants were dried on a SpeedVac at 30°C and reconstituted in 0.5 mL of LC‐matching solvent containing 17.8 μM [2‐^13^C] tyrosine and 39.2 μM [3‐^13^C] lactate (^13^C labeled internal standards were added to the reconstituting solvent in order to monitor LC–MS performance). Samples were transferred into LC vials and placed into a 4°C auto‐sampler for LC–MS analysis.

### 4.7 LC–MS assay

Targeted LC–MS metabolite analysis was performed on a duplex‐LC–MS system composed of two Shimadzu UPLC pumps, CTC Analytics PAL HTC‐xt temperature‐controlled auto‐sampler and AB Sciex 6500+ Triple Quadrupole MS equipped with ESI ionization source (Meador et al., 2020). UPLC pumps were connected to the auto‐sampler in parallel and were able to perform two chromatographic separations independently from each other. Each sample was injected twice on two identical analytical columns (Waters XBridge BEH Amide XP) performing separations in hydrophilic interaction liquid chromatography mode. While one column was performing separation and MS data acquisition in ESI+ ionization mode, the other column was being equilibrated prior to sample injection, chromatographic separation and MS data acquisition in ESI‐ mode. Each chromatographic separation was 18 min (total analysis time per sample was 36 min). MS data acquisition was performed in multiple‐reaction‐monitoring mode. The LC–MS system was controlled using AB Sciex Analyst 1.6.3 software. Measured MS peaks were integrated using AB Sciex MultiQuant 3.0.3 software. In every sample the LC–MS assay detected 158 metabolites, four of which were spiked isotopic reference internal standards. In addition to the study samples, two sets of quality control (QC) samples were used to monitor the assay performance as well as data reproducibility. One QC [QC(I)] consisted of a pooled human serum sample used to monitor system performance and the other QC [QC(S)] consisted of pooled study samples and was used to monitor data reproducibility. Each QC sample was injected per every 10 study samples. The data were highly reproducible, with a median CV of 5.1%.

### 4.8 Metabolomic data analysis

LC–MS peak intensity data from 154 metabolites were log_e_ transformed and then the data within each sample were mean‐centered and scaled to SD = 1. Potential effects of two metabolite extraction batches were removed using the ComBat function in the sva package (Johnson et al., 2007). Principal components were computed on scaled metabolite values. Individual metabolites whose abundance might differ between sensitive and resistant lines were tested by Type III ANOVA with a treatment by phenotype interaction term ($\beta_{T\times P}$), including line as a random effect:

$$\text{Metabolite}\sim \beta_{T}+\beta_{P}+\beta_{T\times P}+1|\text{line}+\varepsilon$$

*p‐*values for each term were FDR‐corrected for multiple comparison. We tested individual metabolites for effects of rapamycin within samples from each phenotype by fitting a mixed model with treatment as a fixed effect, and a random effect of line, with FDR correction.

$$\text{Metabolite}\sim \beta_{T}+1|\text{line}+\varepsilon$$

Pathway enrichment of metabolites with significant treatment effects was performed with the FELLA package (Picart‐Armada et al., 2018). A network graph of 4173 metabolites, 5724 reactions, 770 enzymes, 176 modules, and 138 pathways, was constructed from the KEGG database release 109.0. Of 154 metabolites measured in this study, 133 were mapped to a KEGG identifier. Of these, 49 had treatment effects in sensitive larvae and four had treatment effects in resistant larvae, and both sets were tested for enrichment of the KEGG network using the network diffusion method (Picart‐Armada et al., 2018). The significance of enrichment was assessed by comparison to 10^5^ permutations within the 133 measured compounds. Empirical *p‐*values were adjusted for multiple testing using the FDR approach.

To represent the metabolomic effect of starvation in a single vector, metabolomic data from five replicates of 25 to 38 whole W^1118^ larvae, at 0, 2, 4, 6, or 8 h on PBS‐soaked paper, were provided by Jouandin et al. (2022). We removed metabolites with >1 missing value and imputed the remaining 10 metabolites that had only one missing value using 10‐nearest neighbor mean imputation. We normalized the data of Jouandin et al. (2022) by mean‐centering and scaling by sample. Names of each metabolite in the two datasets were manually matched. Of the 84 intersecting metabolites, nine metabolites had two complementary measurements from both positive and negative ion LC/MS modes in the Jouandin et al data. To estimate the levels of these metabolites, we scaled the data by metabolite and took the mean of each pair of ions for each sample. We then performed PCA on the data from Jouandin et al. and, used non‐linear least squares in the stats R package to fit an intercept (*a* = 20.53), and two shape parameters (*b* = 2.77 and *c* = 10.01) in the model shown below, finding that PC_starvation_ had a strong non‐linear relationship with starvation time (time, *r* ^2^ = 0.91, *p* = 1.3 × 10^−13^):

$${\mathrm{PC}}_{\text{starvation}}=a\;\left(\text{time}/\text{time}+b\right)-c$$

We used loadings of the 84 metabolites on PC_starvation_ to assess the metabolome of rapamycin‐treated larvae compared to control larvae in our study. The interaction between rapamycin treatment and the sensitivity of the larvae on PC_starvation_ was assessed with a mixed model.

$${\mathrm{PC}}_{\text{starvation}}\sim \beta_{T}+\beta_{P}+\beta_{T\times P}+1|\text{line}+\varepsilon$$

## AUTHOR CONTRIBUTIONS

M.B.L. and B.R.H. conceived and designed the experiments, collected, and analyzed data, and wrote the paper. S.Z. and B.Y. helped conceive and design experiments, collected data, and contributed to data analysis. K.H., J.S., V.P., S.T., D.K., H.F., Y.‐C.P., and P.T. collected data. X.Z. and A.W.J. contributed to experimental design and data analysis. D.R. and D.E.L.P. helped conceive and design the experiments, analyze data, and helped write the manuscript. All authors reviewed and approved of final manuscript.

## FUNDING INFORMATION

This work was supported by the National Institutes of Aging (grant AG049494) and by NIH grant 1S10OD021562‐01 to the Northwest Metabolomics Research Center. M.B.L. was supported by the NIH Alzheimer's Disease Training Program (T32AG05235). D.P. was supported in part by USDA cooperative agreement USDA/ARS 58‐8050‐9‐004. A.J. and B.Y. were supported in part by the University of Washingtion Levinson Emerging Scholars Award, and S.Z., K.H., V.P., J.S., A.J. B.Y. and S.T. were supported by the University of Washington Mary Gates Endowment.

## CONFLICT OF INTEREST STATEMENT

The authors declare no competing interests.

## Supporting information


Figure S1.

Table S1.

Table S2.

Table S3.

Table S4.

Table S5.

## Data Availability

Code and data have been deposited at GitHub: https://github.com/ben6uw/Harrison_Lee_et‐al‐2024 and are publicly available as of the date of publication. Additional genetic data used in this analysis are available at: http://dgrp2.gnets.ncsu.edu/data.html. Further information and requests for resources and reagents should be directed to Daniel Promislow (daniel.promislow@tufts.edu).
