# Supplementary material for: Wide‐ranging genetic variation in sensitivity to rapamycin in Drosophila melanogaster
Source: Aging Cell. 2024 Aug 12;23(11):e14292. doi: 10.1111/acel.14292 (PMC11561674; doi:10.1111/acel.14292)
Supplement: Supplementary file 1 — Figure S1. [file ACEL-23-e14292-s001.pdf]

Figure 1.

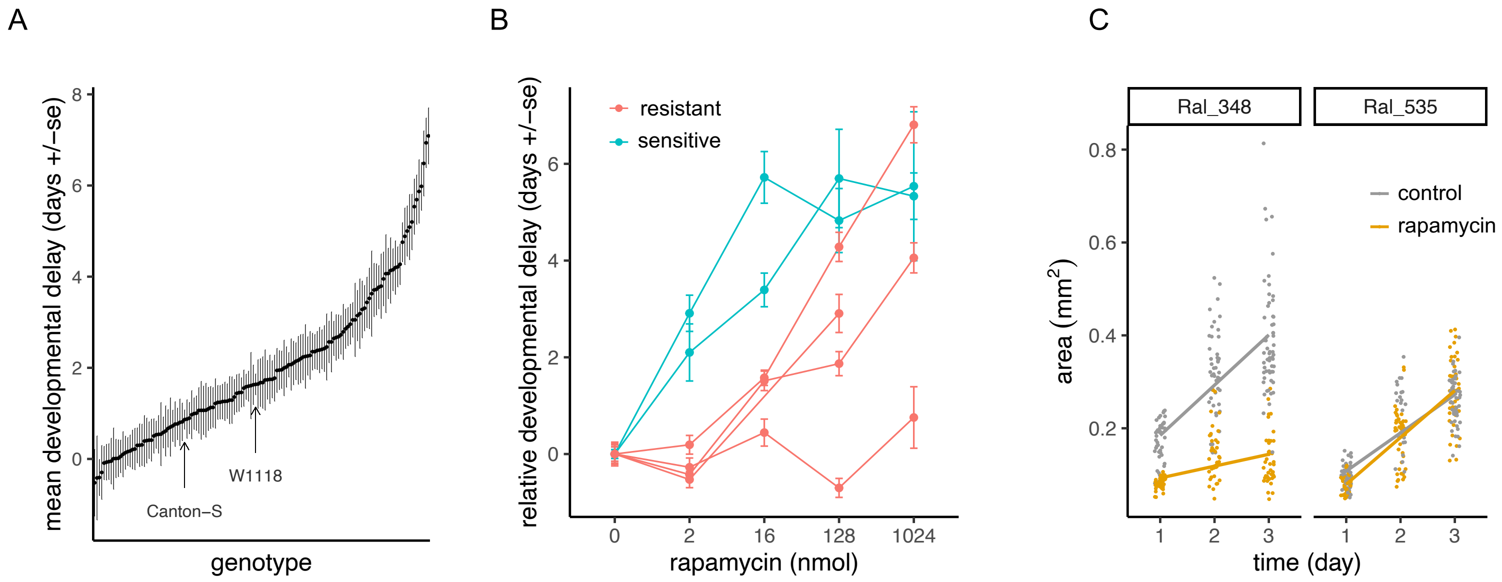

A
mean developmental delay (days +/−se)
8
6
4
2
0
Canton−S
W1118
genotype
B
resistant
sensitive
relative developmental delay (days +/−se)
6
4
2
0
0
2
16
128
1024
rapamycin (nmol)
C
Ral_348
Ral_535
0.8
0.6
0.4
0.2
area (mm²)
control
rapamycin
1
2
3
1
2
3
time (day)

Figure 2.

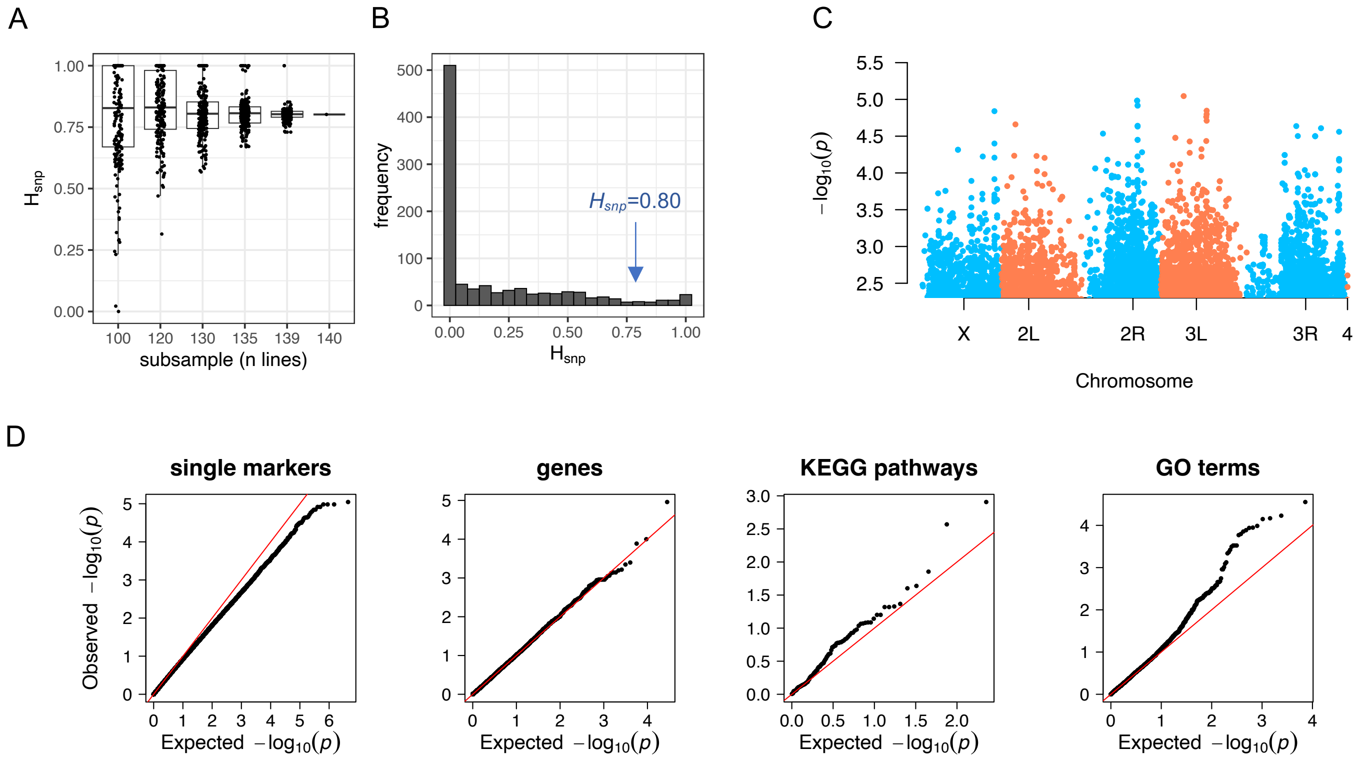

A
B
C
D
1.00
0.75
0.50
0.25
0.00
H_{snp}
100
120
130
135
139
140
subsample (n lines)
500
400
300
200
100
0
frequency
H_{snp}=0.80
0.00
0.25
0.50
0.75
1.00
H_{snp}
5.5
5.0
4.5
4.0
3.5
3.0
2.5
$-\log_{10}(p)$
X
2L
2R
3L
3R
4
Chromosome
single markers
genes
KEGG pathways
GO terms
Observed $-\log_{10}(p)$
Expected $-\log_{10}(p)$

Figure 3.

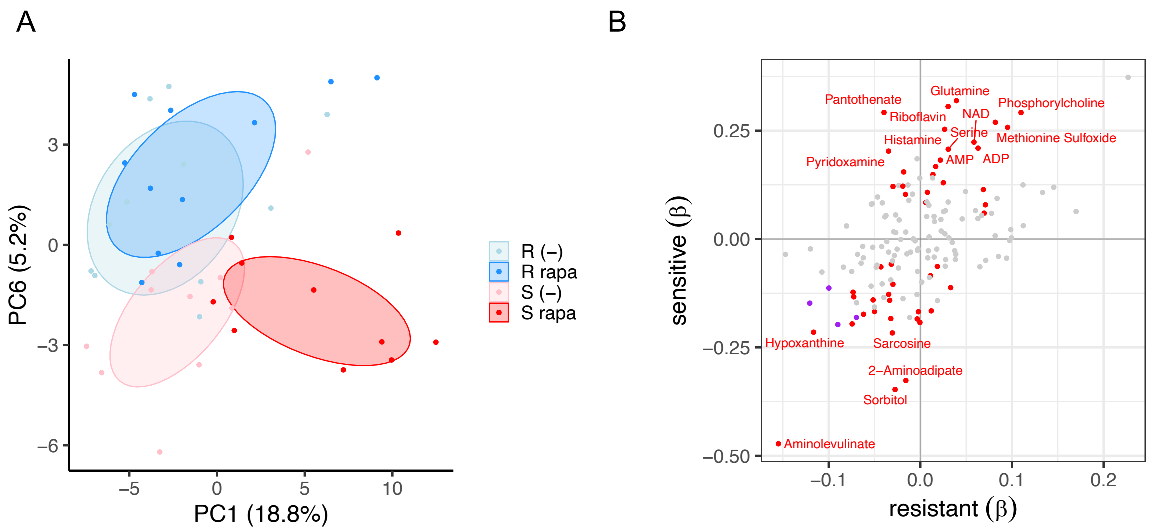
A
B
PC6 (5.2%)
PC1 (18.8%)
3
0
−3
−6
−5
0
5
10
R (–)
R rapa
S (–)
S rapa
sensitive (β)
resistant (β)
0.25
0.00
−0.25
−0.50
−0.1
0.0
0.1
0.2
Pantothenate
Glutamine
Riboflavin
NAD
Phosphorylcholine
Serine
Histamine
Methionine Sulfoxide
AMP
ADP
Pyridoxamine
Hypoxanthine
Sarcosine
2−Aminoadipate
Sorbitol
Aminolevulinate

Figure 4.

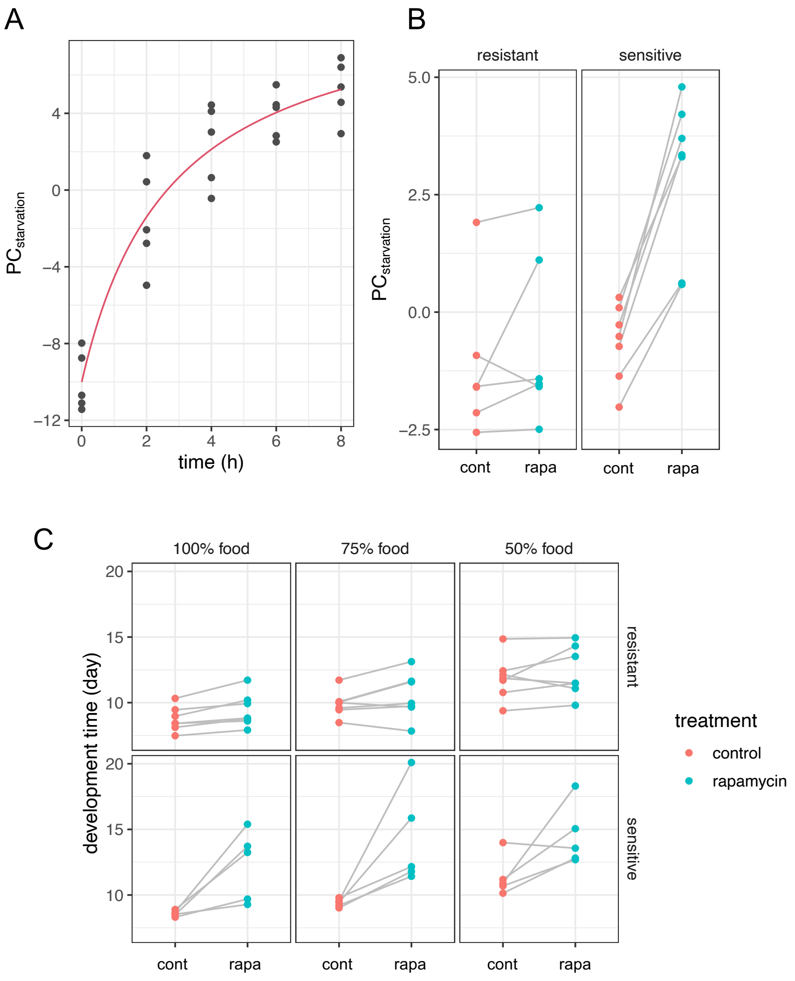

A
PC starvation
time (h)
B
resistant
sensitive
cont
rapa
C
100% food
75% food
50% food
development time (day)
resistant
sensitive
treatment
control
rapamycin

Supplemental Figure 1

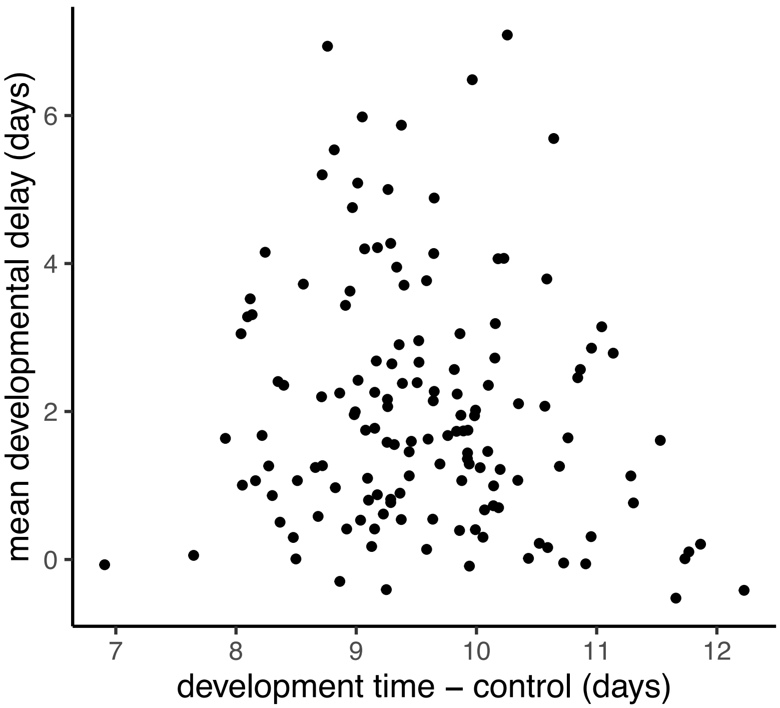

Supplemental Figure 1 Response to rapamycin is not explained by basal development time. The mean developmental delay (mean time on rapamycin – mean time in control) of 140 DGRP lines plotted over the mean control development time. There is no correlation between these two phenotypes, Spearman's ρ=-0.133, P=0.115.

Supplemental Figure 2.

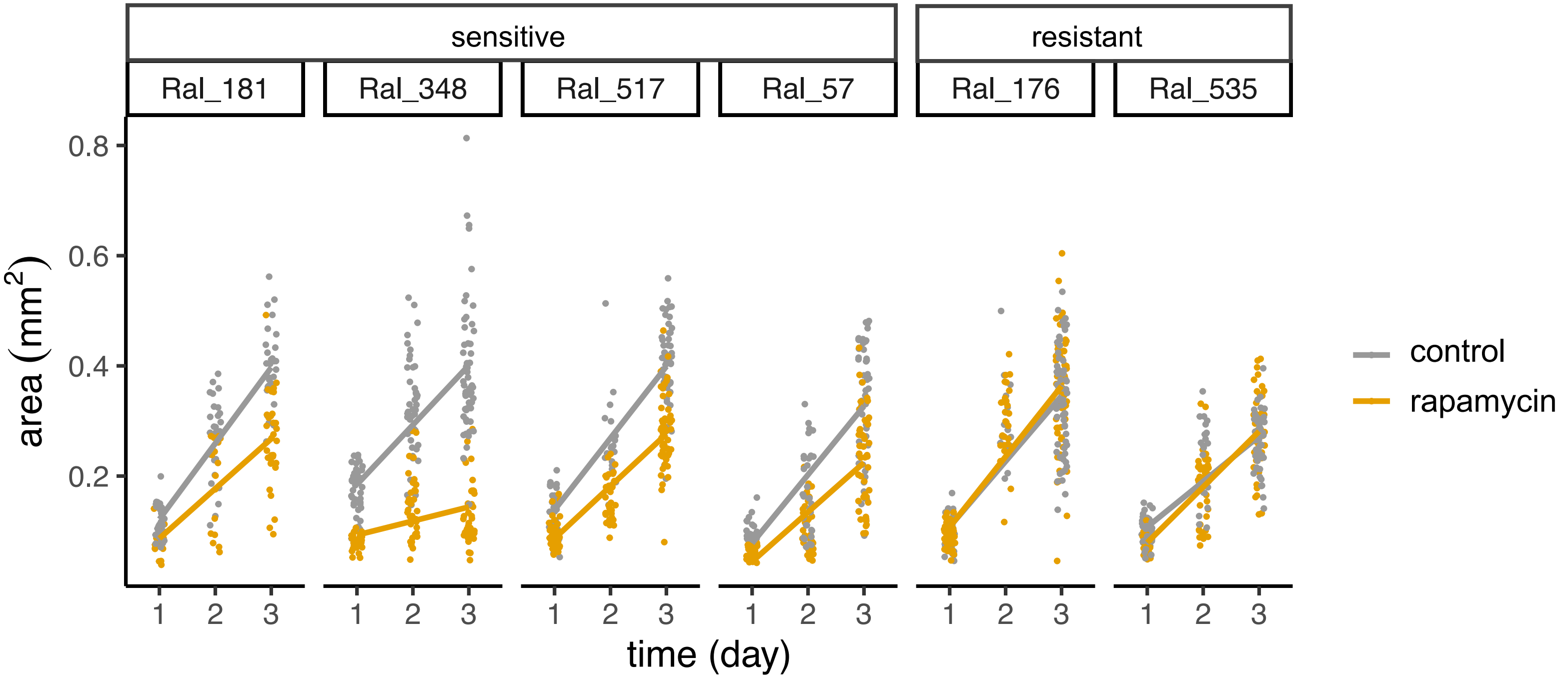

Supplemental Figure 2 Larval growth rate is sensitive to rapamycin. The size (area) of larvae from four lines that are sensitive to rapamycin (mean delay = 6.21±0.63d) and two resistant lines (mean delay =-0.022±0.05d) was monitored over the first 3 days of development on either control food (grey), or food containing 2 nmol rapamycin (yellow). The area of 17 to 67 larva was measured each day from new samples each day. Both the size, as well as the rate of size increase over time were significantly affected by rapamycin in sensitive lines, but not in resistant lines (ANOVA, $P<0.05$).
